# Supplementary material for: Brain-wide presynaptic networks of functionally distinct cortical neurons
Source: Nature. 2025 Feb 26;641(8061):162–72. doi: 10.1038/s41586-025-08631-w (PMC12043506; doi:10.1038/s41586-025-08631-w)
Supplement: Supplementary file 1 — Reporting Summary [file 41586_2025_8631_MOESM1_ESM.pdf]

nature portfolio

Corresponding author(s): Ana R Inacio and Soohyun Lee

Last updated by author(s): Oct 13, 2024

Reporting Summary

Nature Portfolio wishes to improve the reproducibility of the work that we publish. This form provides structure for consistency and transparency in reporting. For further information on Nature Portfolio policies, see our Editorial Policies and the Editorial Policy Checklist.

Statistics

For all statistical analyses, confirm that the following items are present in the figure legend, table legend, main text, or Methods section.

n/a	Confirmed	
☐	☒	The exact sample size (*n*) for each experimental group/condition, given as a discrete number and unit of measurement
☐	☒	A statement on whether measurements were taken from distinct samples or whether the same sample was measured repeatedly
☐	☒	The statistical test(s) used AND whether they are one- or two-sided<br>*Only common tests should be described solely by name; describe more complex techniques in the Methods section.*
☐	☒	A description of all covariates tested
☐	☒	A description of any assumptions or corrections, such as tests of normality and adjustment for multiple comparisons
☐	☒	A full description of the statistical parameters including central tendency (e.g. means) or other basic estimates (e.g. regression coefficient) AND variation (e.g. standard deviation) or associated estimates of uncertainty (e.g. confidence intervals)
☐	☒	For null hypothesis testing, the test statistic (e.g. *F*, *t*, *r*) with confidence intervals, effect sizes, degrees of freedom and *P* value noted<br>*Give P values as exact values whenever suitable.*
☒	☐	For Bayesian analysis, information on the choice of priors and Markov chain Monte Carlo settings
☐	☒	For hierarchical and complex designs, identification of the appropriate level for tests and full reporting of outcomes
☐	☒	Estimates of effect sizes (e.g. Cohen's *d*, Pearson's *r*), indicating how they were calculated

Our web collection on statistics for biologists contains articles on many of the points above.

Software and code

Policy information about availability of computer code

Data collection	Two-photon calcium imaging data was acquired using a commercially available system, including software (PrarieView, 5.4 and 5.5), from Bruker.<br>Behavioral data was acquired using custom-written codes in LabVIEW (2017 and 2019, National Instruments) and the high speed digital video recording software StreamPix (7 and 9, NorPix).<br>Behavioral and neuronal data were synchronized through the Bruker system.<br>Histological images were acquired using Neurolucida (v2019-2021, MBF Bioscience), and software from Nikon (NIS-Elements 5.20.01) and Zeiss (ZEN 2.3 and 3.1), as specified throughout the Methods.
Data analysis	Two-photon calcium images were processed using Suite2p (0.10.1), in Python (Pachitariu et al., BioRxiv, 2017; reference provided in article).<br>Behavioral video recordings were preprocessed using custom MATLAB (R2020a and R2022a) routines.<br>Neural and behavioral signals were analyzed using custom-written MATLAB (R2020a and R2022a) or Python (3.7.6 and 3.11.5) routines, including the Scikit-learn library (1.3.0; Pedregosa et al., Journal of Machine Learning Research, 2011; reference provided in article).<br>Histological analyses were performed using NeuroInfo (2019-2023, MBF Bioscience), as well as ImageJ (1.52p, NIH).<br>Analysis of presynaptic networks was performed using Python (3.8.19), including the following library: POT Python Optimal Transport library (0.9.3, Villani, Topics in Optimal Transportation, 2021; reference provided in article).<br><br>The code for analysis is available at: https://github.com/NIMH-FNC/Brain-wide-presynaptic-networks.

For manuscripts utilizing custom algorithms or software that are central to the research but not yet described in published literature, software must be made available to editors and reviewers. We strongly encourage code deposition in a community repository (e.g. GitHub). See the Nature Portfolio guidelines for submitting code & software for further information.

Data

Policy information about availability of data

All manuscripts must include a data availability statement. This statement should provide the following information, where applicable:

- Accession codes, unique identifiers, or web links for publicly available datasets
- A description of any restrictions on data availability
- For clinical datasets or third party data, please ensure that the statement adheres to our policy

The datasets are available from the corresponding authors upon reasonable request.

Research involving human participants, their data, or biological material

Policy information about studies with human participants or human data. See also policy information about sex, gender (identity/presentation), and sexual orientation and race, ethnicity and racism.

Reporting on sex and gender	N/A
Reporting on race, ethnicity, or other socially relevant groupings	N/A
Population characteristics	N/A
Recruitment	N/A
Ethics oversight	N/A

Note that full information on the approval of the study protocol must also be provided in the manuscript.

Field-specific reporting

Please select the one below that is the best fit for your research. If you are not sure, read the appropriate sections before making your selection.

☒ Life sciences ☐ Behavioural & social sciences ☐ Ecological, evolutionary & environmental sciences

For a reference copy of the document with all sections, see nature.com/documents/nr-reporting-summary-flat.pdf

Life sciences study design

All studies must disclose on these points even when the disclosure is negative.

Sample size	We did not use statistical methods to predetermine sample size. Our sample sizes were estimated based on previous publications using a similar methodology (Velez-Fort et al., Neuron, 2014; Wertz et al., Science, 2015; Rossi et al., Nature, 2020).
Data exclusions	Inclusion and exclusion criteria were established a priori. Experiments in which TTX did not silence neuronal activity over the entire FOV within 15-20 min, indicative of limited drug diffusion, were excluded (n = 2 mice). Brains containing less than 100 presynaptic cells were excluded from analysis (n = 1).
Replication	Results were reliably replicated across mice, as evidenced by the individual data points included in the Figures. Analytical routines and statistical tests were established using a subset of the data and then applied to entire datasets.
Randomization	Animals in test and control groups were littermates and randomly selected. In in vivo neuropharmacological experiments, receptor blocker application session sequences were randomly assigned across animals. In optogenetic experiments, light pulses (1-1.5 s) were randomly provided during the recording session.
Blinding	All data were acquired according to standard protocols, batch processed using the same codes, and independently processed and analyzed by analysts blinded to the experimental groups. In addition, manual and semi-automatic routines involving ROI curation in two-photon calcium imaging data, histological analysis, and classification of presynaptic neurons were done blindly, as specified in the Methods.

Reporting for specific materials, systems and methods

We require information from authors about some types of materials, experimental systems and methods used in many studies. Here, indicate whether each material, system or method listed is relevant to your study. If you are not sure if a list item applies to your research, read the appropriate section before selecting a response.

Materials & experimental systems

n/a	Involved in the study
☐	☒ Antibodies
☒	☐ Eukaryotic cell lines
☒	☐ Palaeontology and archaeology
☐	☒ Animals and other organisms
☒	☐ Clinical data
☒	☐ Dual use research of concern
☒	☐ Plants

Methods

n/a	Involved in the study
☒	☐ ChIP-seq
☒	☐ Flow cytometry
☒	☐ MRI-based neuroimaging

Antibodies

Antibodies used	Antibodies were used to detected red fluorescent proteins (RFP) and γ-Aminobutyric acid (GABA) in formaldehyde-fixed coronal brain sections.<br>Primary antibodies and respective dilutions:<br>- Chicken anti-RFP (600-901-379, Rockland), 1:500,<br>- Rabbit anti-GABA (A2052, Sigma), 1:500 .<br>Secondary antibodies and respective dilutions:<br>- Goat anti-chicken IgY-Alexa Fluor 555 (A21437, Thermo Fisher Scientific), 1:200,<br>- Goat anti-rabbit IgG-Alexa Fluor 647 (A21245, Thermo Fisher Scientific), 1:200 .
Validation	We used primary antibodies that have been validated by the manufacturers and are widely used in the field, as reflected by the large number of product citations including similar histological applications.<br><br>Anti-RFP manufacturer notes.<br>Applications. ELISA, SDS-PAGE, WB, FC, IF, IHC.<br>Purity/Specificity. RFP Antibody was prepared from egg yolks by a multi-step process which includes filtration, delipidation, salt fractionation and extensive dialysis against the buffer stated above. RFP Antibody was tested by western blot.<br><br>Anti-GABA manufacturer notes.<br>Technique(s). Dot blot: 1:10,000; immunohistochemistry (formalin-fixed, paraffin-embedded sections): 2.5 µg/mL using rat cerebellum.<br>General description. Anti-GABA is produced in rabbit using GABA-BSA as the immunogen. The antibody is isolated from antiserum by immunospecific methods of purification. Antigen specific affinity isolation removes essentially all rabbit serum proteins, including immunoglobulins which do not specifically bind to GABA.<br>Application. Expression of GABA in neucortical cells harvested from the brains of E19 day old rat embyros was detected by immunofluorescence using rabbit anti-GABA antibody. Triple IF staining was performed with the anti-GABA antibody and two anti-GAD antibodies. Expression of GABA was analyzed in cells isolated from the pallium of various animals including rats, mice, rabbits, guinea pigs, and lizards by immunohistochemistry. IHC was performed using rabbit anti-GABA antibody at 1:1000 diluted in a solution of 0.01M PBS ph 7.4 + 0.5% triton-x100.<br><br>Further details on the validation procedures and product citations can be found at:<br>- https://www.rockland.com/categories/primary-antibodies/rfp-antibody-600-901-379 (49 references),<br>- https://www.sigmaaldrich.com/US/en/product/sigma/a2052 (562 references).

Animals and other research organisms

Policy information about studies involving animals; ARRIVE guidelines recommended for reporting animal research, and Sex and Gender in Research

Laboratory animals	We used the following transgenic mouse lines: Emx1-IRES-Cre (JAX 005628), tetO-GCaMP6s (JAX 024742), and CaMK2a-tTA (JAX 007004). We performed experiments on 10 Emx1-IRES-Cre, 71 tetO-GCaMP6s;CaMK2a-tTA, and 3 GCaMP6s+;CaMK2atTA- mice that were generated from the parental lines described above. Mice were 12-24 weeks old at the experimental endpoint. They were housed in groups, in individually ventilated and enriched laboratory cages, in climate-controlled rooms (T, 22 ºC; humidity, 45%), under a reverse 12 h light - 12 h dark cycle, and with ad libitum access to water and food.
Wild animals	No wild animal was used in the study.
Reporting on sex	We used both male and female mice (females, 45%). This study was not designed to identify sex-specific differences. Our sample sizes are not suitable to account for sex-dependent differences in the stability of the neuronal activity patterns, effect of ascending neuromodulatory systems or presynaptic connectivity motifs in the context of behavioral state. Neverthless, overall, the percentage of Movup and Movdown neurons was similar between the male and female groups (30 ± 11% vs. 33 ± 11 %, n = 26 vs. 21 animals, respectively, P = 0.27, Wilcoxon rank-sum test). Similarly, the percentage of sensory-responsive neurons did not differ between males and females (13 ± 5.9% vs. 12 ± 6.1%, n = 19 vs. 14 animals, respectively; P = 0.57, Wilcoxon rank-sum test).
Field-collected samples	No field-collected samples were used in the study.

Ethics oversight	All animal procedures were conducted in accordance with a protocol approved by the National Institutes of Health Institutional Animal Care and Use Committee (IACUC), Bethesda, MD, USA, and complied with the Public Health Service Policy on Humane Care and Use of Laboratory Animals and the Guide for the Care and Use of Laboratory Animals.

Note that full information on the approval of the study protocol must also be provided in the manuscript.
